# A metagenomic survey of soil microbial communities along a rehabilitation chronosequence after iron ore mining

**DOI:** 10.1038/sdata.2019.8

**Published:** 2019-02-12

**Authors:** Markus Gastauer, Mabel Patricia Ortiz Vera, Kleber Padovani de Souza, Eder Soares Pires, Ronnie Alves, Cecílio Frois Caldeira, Silvio Junio Ramos, Guilherme Oliveira

**Affiliations:** 1 Instituto Tecnológico Vale, Rua Boaventura da Silva, 955, Bairro: Nazaré, CEP 66055-090, Belém, PA, Brazil; 2 Universidade Federal do Pará, Programa de Pós-Graduação em Genética e Biologia Molecular, Rua Augusto Corrêa, 01, Guamá, CEP 66075-110, Belém, PA, Brazil; 3 Universidade Federal do Pará, Programa de Pós-Graduação em Ciência da Computação, Rua Augusto Corrêa, 01, Guamá, CEP 66075-110, Belém, PA, Brazil

**Keywords:** Metagenomics, Genomics, Environmental chemistry

## Abstract

Microorganisms are useful environmental indicators, able to deliver essential insights to processes regarding mine land rehabilitation. To compare microbial communities from a chronosequence of mine land rehabilitation to pre-disturbance levels from references sites covered by native vegetation, we sampled non-rehabilitated, rehabilitating and reference study sites from the Urucum Massif, Southwestern Brazil. From each study site, three composed soil samples were collected for chemical, physical, and metagenomics analysis. We used a paired-end library sequencing technology (NextSeq 500 Illumina); the reads were assembled using MEGAHIT. Coding DNA sequences (CDS) were identified using Kaiju in combination with non-redundant NCBI BLAST reference sequences containing archaea, bacteria, and viruses. Additionally, a functional classification was performed by EMG v2.3.2. Here, we provide the raw data and assembly (reads and contigs), followed by initial functional and taxonomic analysis, as a base-line for further studies of this kind. Further investigation is needed to fully understand the mechanisms of environmental rehabilitation in tropical regions, inspiring further researchers to explore this collection for hypothesis testing.

## Background & Summary

In many countries, the environmental rehabilitation of mine lands as close as possible to its pre-disturbance levels is a legal requirement to reduce net losses of biodiversity and ecosystem functions^1,2^. It is necessary to monitor rehabilitating sites to meet targets of environmental licensing agencies^2^. To date, there is no consensus on the best indices available from science to evaluate the monitoring process^3^. Therefore, multidisciplinary approaches aiming at providing such parameters have been proposed recently^4,5^.

Besides vegetation or fauna surveys^6^, the examination of microbial communities can detect environmental alterations in short time scales^7^, thus able to deliver insights about the fulfillment of rehabilitation targets^8,9^. Metagenomic approaches provide insights into environmental variations^10–12^, detecting the diversity of microorganisms in rehabilitating habitats^13^. Comparing the composition of microbial communities from rehabilitating communities to preserved reference sites may thus contribute to the evaluation of rehabilitation success in mine lands^14,15^.

In Brazil, one of the world’s leading raw iron export nations^16^, iron ore deposits occur in open-cast mines in different regions. Ferriferous savanna ecosystems named ‘c*angas’*^17,18^ cover the deposits in the Iron Quadrangle (Minas Gerais), the Carajás mountains (Pará), the Caetité region (Bahia), and the Urucum Massif (Mato Grosso do Sul). Due to particular environmental conditions such as high concentrations of metal ions, especially iron, high radiation, elevated temperatures, and ample rainfall seasonal amplitudes, these diverse and endemic ecosystems^19–21^ are considered hotspots of biodiversity^17,22^. Besides the storage of unique genetic resources for therapeutic purposes^23^ or the remediation of contaminated areas^24,25^, rupestrian *canga* ecosystems provide many ecosystem services^26^.

Impacted by iron ore extraction^27^ reshaping entire landscapes^28^ by the removal of ore and mining wastes, the environmental rehabilitation of the impacted ecosystems is desired aiming at the preservation of biotic resources and ecosystem services for future generations. Insights to composition, diversity and functional characterization of microbial soil communities along environmental rehabilitation gradients are useful variables for measuring the success of rehabilitation, able to provide valuable feedback to improve the rehabilitation practice.

The goal of this study was to identify changes in microbial community composition, diversity and functional processes resulting from mine land rehabilitation and compare to pre-disturbance levels from references sites covered by native *canga* vegetation. We sampled three study sites before rehabilitation efforts, seven study sites spanning different rehabilitation stages and three reference *canga* sites associated with two iron ore mines from Corumbá (Urucum Massif). Environmental rehabilitation comprises topographic reformulation after removal of the iron ore, liming, fertilization and the application of biomass before native *canga* species are seeded or planted.

At each study site, we installed three plots of 10 × 10 m; in each plot, a composed soil sample was collected (depth 0–2 cm) for metagenomics analysis. An additional sample (depth 0–10 cm) was collected for physical and chemical analysis. In this study, we applied a paired-end sequencing technology (NextSeq 500 Illumina) after DNA extraction, purification and amplification to construct metagenomic libraries. The Illumina reads were assembled using MEGAHIT. Subsequently, nucleotide sequences coding for proteins (CDS), were extracted from assemblies. Functional and taxonomic classification of coding DNA sequences (CDS) was performed using EMG and Kaiju.

Here, we provide the complete metagenomic data set, without detailed analysis of results or discussion to highlight its outstanding comprehensive view into soil microbial communities from the rehabilitation of a *canga* ecosystem occurring in Southwestern Brazil. We furthermore present the annotated metagenome assembly, containing taxonomic and functional classification as well as chemical soil properties (i.e., pH, cation exchange capacity, organic matter contents, micro- and macro nutrient as well as aluminum availability) and soil texture. The present collection is the first high-throughput sequencing-based survey from non-rehabilitated and reference sites as well as sites under rehabilitation after iron ore mining from a tropical region, thus representing base-line data for further studies of this kind. With its publication, researchers can explore this collection for hypothesis testing related to environmental rehabilitation in tropical regions, especially after mining activities. The consistency in experimental design, sequencing methodology and sample sources ensures the value of this collection for on-going studies about environmental rehabilitation after anthropogenic impacts, in particular, those about mine land rehabilitation.

## Methods

### Experimental design

**Figure 1:**
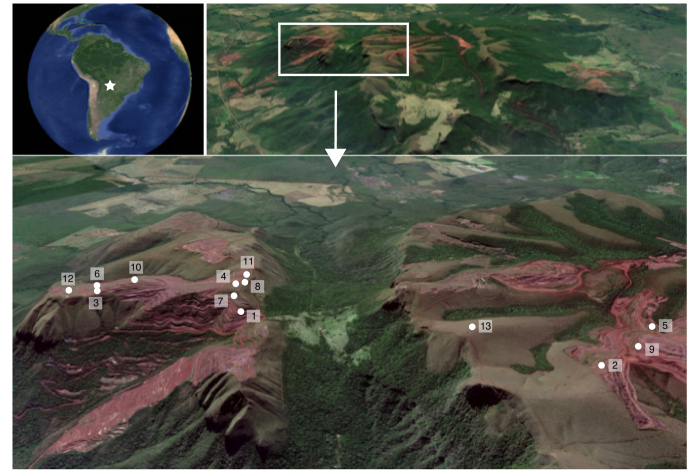
Map of geographical position of the study sites in the Urucum Massif, Corumbá, Mato Grosso do Sul, Brazil. 1. Rampa Nova, 2. Mina 5, 3. PRAD 45 C, 4. Piscinão 5. Mina Cateto, 6. PRAD 45 A, 7. Mina Escarpa, 8. Secção 10I, 9. Mina 5 N, 10. PRAD 45B, 11. Reference A, 12. Reference B, and 13. Reference C.

Data were collected in October 2016 in 13 study sites from open-cast iron ore mines situated in the Urucum Massif, Mato Grosso do Sul, Brazil (Fig. 1). The altitude of the massif varies between 600 and 1,065 m a.s.l. With a mean annual temperature of 25 °C and mean annual precipitation of 1,070 mm^29^, the climate of the region corresponds a tropical warm, savanna climate (Aw in the Koppen classification), characterized by dry winters and rainy summers. The natural vegetation is a mosaic of seasonal deciduous and semi-deciduous forests on slopes and near watercourses. Furthermore, different savanna formations, ranging from arborized physiognomies to treeless grasslands stock on the upper parts of the massif ^30^.

Iron ore mining in the region is restricted to the outcrops of ferruginous jaspilites and fixed hematites from the Santa Cruz Formation^31^ and begins with the suppression of vegetation and removal of topsoil layers. Environmental rehabilitation after mining includes topological reformulation, topsoil application, liming and fertilization of mine soils. Organic matter originating from suppressed areas is added. The rehabilitation targets are native open savanna formations, i.e., pre-mining formations on ironstone outcrops. Thus, plants rescued from suppressed areas and seedlings of native species produced in a tree nursery are planted to trigger environmental rehabilitation of mine lands. Additionally, seed mixtures of native species collected in the vegetation remnants were applied. On-demand, further activities, such as re-plantation of seedlings, further applications of seeds, and combating alien invasive species, were executed.

Study sites comprise three bare soil areas immediately before rehabilitation activities are carried out, seven sites from different rehabilitating stages (two-, three- and six-year-old stands) as well as three reference sites covered by native vegetation, i.e., open savanna formations (Table 1). At each study site, three plots (10 × 10 m) were installed in homogeneous vegetation without signs of external disturbances.

Two mixed soil samples were collected from each plot. For each sample, the substrate from five homogeneously distributed sampling points within each plot was mixed. The first sample collected at a depth of 0–10 cm was air dried and submitted to analysis of chemical properties and texture. The pH in water (pH(H2O)) and in potassium chloride (pH(KCl)), organic matter (OM), available phosphorus (P), potassium (K), sulfur (S), calcium (Ca), magnesium (Mg), aluminum (Al), boron (B), zinc (Zn), iron (Fe), manganese (Mn) and copper (Cu) concentrations as well as effective cation exchange capacity (ECEC) of the samples were determined following standardized protocols^32^. Soil texture was detected by particle-size distribution analysis using the pipette method.

A mixed superficial soil sample (depth 0–2 cm) was collected for metagenomics analysis from each plot. Immediately after collection, soil samples were cooled in a fridge to avoid DNA degeneration. At the lab, the samples were stored in a freezer of −80 °C until analysis.

### DNA extraction and shotgun sequencing

From 250 mg soil from each sample, total DNA was extracted using the PowerSoil DNA Isolation Kit (Mobio Laboratories, USA) following the manufacturer’s instructions. DNA samples were quantified using Qubit 3.0 fluorometer (Thermo Fisher Scientific Inc.).

Shotgun metagenomic paired-end libraries were then constructed from 50 ng of pure DNA. For that, samples were subjected to a random enzymatic fragmentation in which the DNA was simultaneously fragmented and bound to adapters using the QXT SureSelect kit (Agilent Technologies). The fragmented DNA was purified using AmPure XP beads (Beckman Coulter) and subjected to an amplification reaction using primers complementary to the Illumina flowcell adapters. Amplified libraries were again purified using AmPure XP beads (Beckman Coulter), quantified using the Qubit 3.0 Fluorometer (Thermo Fisher Scientific Inc.) and checked for fragments size in the 2100 Bioanalyzer (Agilent Technologies®) using a High Sensitivity DNA kit (Agilent Technologies).

After that, the libraries were adjusted to a concentration of 4 nM, pooled, denatured and diluted to a running concentration of 1.8 pM. The sequencing run was performed in the NextSeq 500 Illumina platform using a NextSeq 500 v2 kit high-output with 150 cycles.

### Genome assembly, taxonomic and functional classification

**Figure 2:**
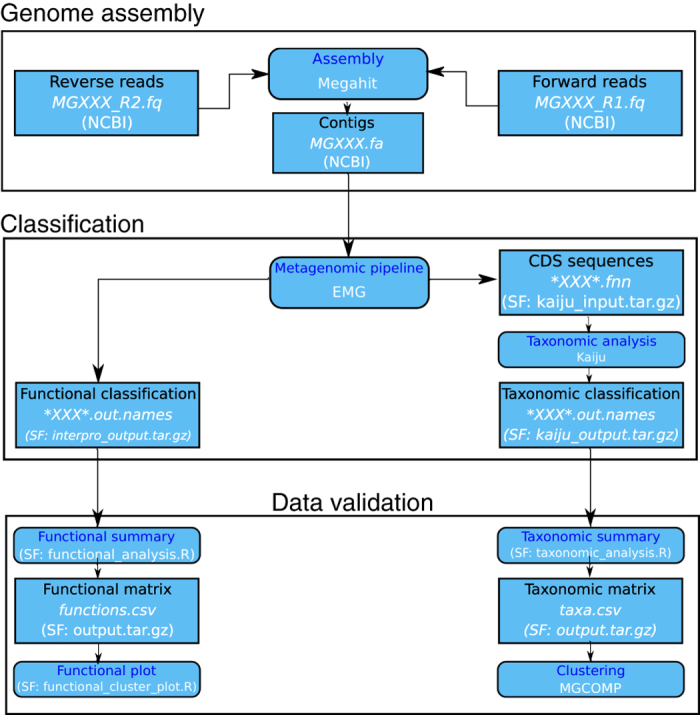
Workflow of genome assembly, functional and taxonomic classification and data validation applied in this study. Rounded rectangles symbolize processes containing descriptions and tools, and rectangles represent input and/or output files enclosing a brief description, file name (^∗^xxx^∗^ is a placeholder for sample ID) and format, as well as their localization. CDS stands for coding DNA sequences. NCBI indicates that files are available from NCBI (Data Citation 1), whereas SF indicates the corresponding files were deposited in Open Science Framework (Data Citation 2).

The Illumina paired-end reads were assembled using MEGAHIT v1.1.2^33^, using default parameters (Fig. 2). Contigs were output in the fasta format.

Using a locally installed EMG v2.3.2 pipeline^34^, coding DNA sequences (CDS) were extracted from contigs output as .fnn files. Furthermore, the pipeline produces the functional classification output as .ipr files. Subsequently, the taxonomic classification was performed on CDS using Kaiju v.1.4.4 (running mode: greedy, with up to 5 substitutions; minimum match: 12; minimum match score: 70)^35^. As reference database, we used the non-redundant NBCI BLAST protein sequences (access on December, 8^th^, 2016, containing 81 M protein sequences from Bacteria, Archaea, and Viruses). We estimated average coverage as the fraction of the observed microbial community covered by the NBCI BLAST protein sequence by package Nonpareil v3.3.3^36^, using forward reads with quality scores greater than Q20, as recommended by the tool.

### Cluster analysis

For data validation, taxonomic and functional counting matrices were generated. Differences in entire microorganism richness, i.e., the taxonomic matrix containing all CDS identified until genus level, between non-rehabilitated, rehabilitating and reference study sites were outlined using one-way ANOVA followed by post-hoc Tukey HSD tests after checking for normality and homogeneity of variance. Diversity was estimated as Shannon’s diversity index H’, using package vegan v2.5-2^37^ in R Environment.

We used package pvclust v2.0^38^ in R Environment v3.4.1^39^ to compute the clusters from the taxonomic counting matrix, considering genus-level predictions from Kaiju. Cluster consistency was tested using the approximately unbiased (au) and the bootstrap probability (bp) statistics^40^. Both statistics return p-values ranging from 0 to 1, where 0 represents a weak consistency and 1 represents a strong consistency for all formed clusters. As au is a better approximation to unbiased p-value than bp, we considered only with au values larger than 0.95, which represents a strong similarity between the grouped samples.

Finally, an integral analysis of taxonomy was performed by MGCOMP^41^ to observe the relationship among sample profiles and sites. In order to reduce the influence of rare organisms in this analysis, we considered only the top 30 most abundant genera for each sample, which corresponds to the smallest number of genera covering 50% of the analyzed sequences. Based on these top 30 genera, we performed a two-level clustering of all identified genera for this analysis. In the first level, the samples that showed similar genus abundances were grouped and in the second level, a second grouping was carried out in each cluster considering only the samples belonging to the respective group. After the grouping, the genera present in all first level groupings (denominated core taxa), the genera present exclusively in each of the first level groupings (denominated exclusive taxa) and the other genera (denominated neutral taxa) were identified.

## Data Records

The raw nucleotide sequences of 1,192,347,558 reads and 2,608,990 contigs extracted from 34 soil samples were deposited as fastq and fasta files at NBCI (Data Citation 1 and Table 2 (available online only)). As required, fastq files contain four lines for each read, that is an identifier of the read, the nucleotide sequence, the placeholder ‘ + ’ for optional annotations (not used here) and the Phred quality score of each nucleotide. fasta files are composed of two elements for each contig, an identifier and the sequence of the contig.

Further data were deposited in Open Science Framework (Data Citation 2). Here, the “supplementary” folder contains quality reports for forward and reverse reads from each sample as well as chemical and physical soil properties. Soil properties are furnished as comma delimited .csv file, named SoilSamples.csv. Read quality reports contains 12 section entitled 1) Basic Statistics, 2) Per base sequence quality, 3) Per tile sequence quality, 4) Per sequence quality scores, 5) Per base sequence content, 6) Per sequence GC content, 7) Per base N content, 8) Sequence Length Distribution, 9) Sequence Duplication Levels, 10) Overrepresented sequences, 11) Adapter Content and 12) Kmer Content. The file README.txt, available in the same folder, contains a brief explanation for each section.

Additionally, the “cluster_analysis” folder contains three subordinated folders. The “inputs” folder contains files regarding CDS detected within assembled contigs whereas the “output” folder contains the taxonomic and the functional classification that were used to generate counting matrices by the corresponding scripts, deposited in the “script” folder.

The “inputs” folder contains three zipped files. First, kaiju_input.tar.gz contains a file for each sample with all identified CDS. The file lists CDS identifiers and their sequences. Second, kaiju_output.tar.gz contains the taxonomic classification for each CDS, stored as individual, tab-delimited files for each sample. An upper case letter indicates the success of taxonomic classification (U is unclassified, C is classified) and is followed by the CDS identifier, the NCBI taxonomy ID for the identified taxon and a string showing taxonomic identification containing domain, phylum, class, order, family, genus and species, separated by semicolons, for each CDS. The identifier is composed of CDS ID, containing the contig ID as well as the initial and final nucleotide positions of the CDS within the contig, all of them joined by underlines to a single string. The interpro_output.tar.gz contains the functional classification. Individual comma-delimited files (.csv) contains the enzyme list detected within each sample. Each file is composed of three columns containing an identifier, the name of the protein as well as the number of occurrences within the analyzed sample.

The “output” folder contains three comma separated files within a zipped folder (output.tar.gz). The files correspond to the expected taxonomic (taxa.csv) and the functional matrices (functions.csv). Additionally, taxa_30.csv shows the taxonomic matrix for the 30 top genera only.

Furthermore, five R scripts used to produce the taxonomic matrix (taxonomic_analysis.R), plot samples clustered by taxonomic composition (taxonomic_cluster_plot.R), plot taxonomic composition of each sample (taxonomic_stacked_plot.R), produce the functional matrix (functional_analysis.R) and to plot samples clustered by functions (functional_cluster_plot.R) are available in the “scripts” folder.

## Technical Validation

Altogether, 2,166,372 CDS were detected. A total of 2.064 genera were present in 1,290,491 CDS, among them 127 archaea, 1,853 bacteria, and 84 virus genera. Richness varies from 739 to 1,894 within samples (Table 3). 273,799 CDS (12.64% of all CDS) remain completely unclassified, and for an additional 875,881 CDS (40.43% of all CDS), only partial matches are available. Functional classification of identified contigs distinguished 10,913 proteins.

**Figure 3:**
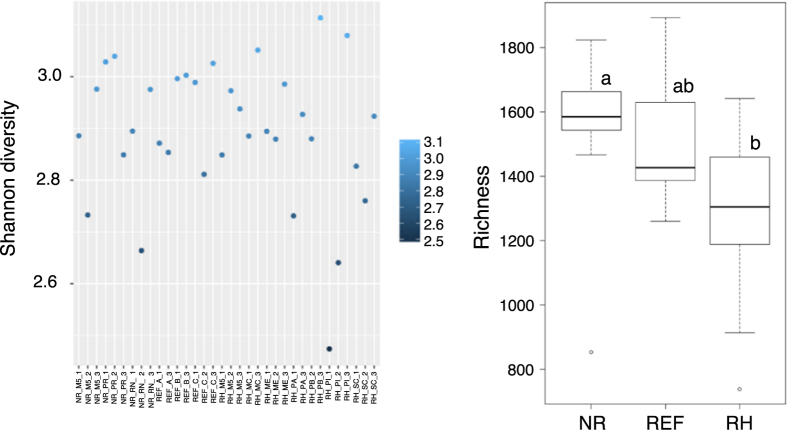
Shannon diversity of each of the 34 samples (left) and boxplot of species richness, separated by non-rehabilitated (NR), rehabilitating (RH) and reference study sites (REF). Different letters in the same boxplot meant significant difference at 0.05 level according to a post-hoc Tukey HSD test. Although no significant difference in richness values between REF to NR and RH, we observed a significant difference between NR to RH.

**Figure 4:**
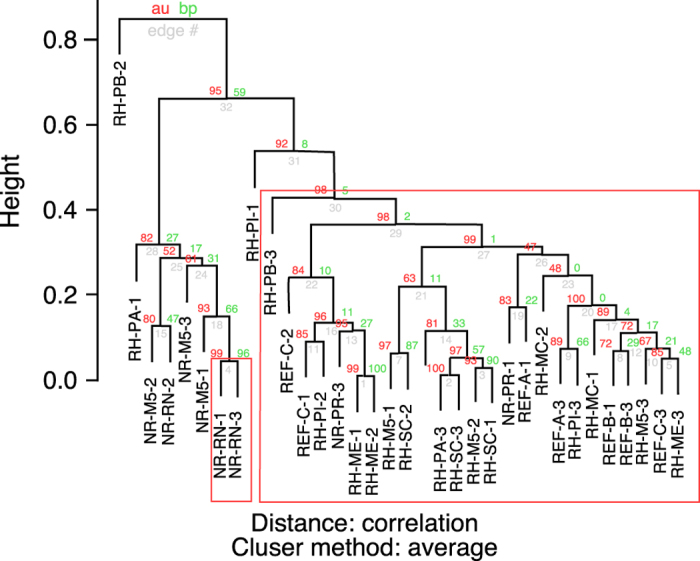
Clustering of samples from non-rehabilitated (NR), rehabilitating (RH) and reference study sites (REF) from Corumbá iron ore mines, Mato Grosso do Sul, Brazil, based on taxonomic counting matrix. We considered only clusters with approximately unbiased clustering statistics (au) larger than 0.95, which represents a strong similarity between the grouped samples.

All micro-organism diversity within samples (measured on genus level) varied from 4.5 to 5.5 (Fig. 3) and was significantly higher in non-rehabilitated than in rehabilitating study sites (ANOVA, F = 4.137, p = 0.0255, Fig. 3). Significant differences in community composition were detected. First, the cluster analysis separated the samples into two clusters. The larger cluster groups samples from rehabilitating and reference sites, whereas samples from non-rehabilitated sites were grouped outside (Fig. 4).

**Figure 5:**
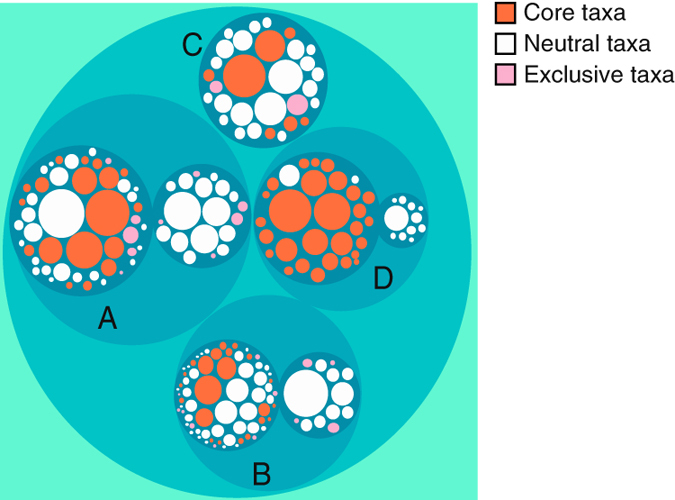
Graphical representations of integrated taxonomy analysis performed by MGCOMP, containing a two-level grouping of all identified genera. Different clusters A, B, C, and D as well as their subclusters, represented as dark blue circles, are composed of different numbers of samples and contain different amounts of core (i.e., present in all first level groupings), exclusive (i.e., occurrence restricted to first level grouping) and neutral (others) genera as shown in Table 4.

Additionally, the complete analysis of taxonomy separated the dataset into four groups by taxonomic profile, three of them divided into subgroups (Fig. 5). As shown in Table 4, samples from all three treatments (non-rehabilitated, rehabilitating and reference sites) were clustered in groups A and B, while a single reference sample forms group D and group C is composed exclusively of non-rehabilitating samples. All analysis carried out here show that taxonomic composition of microorganism communities from rehabilitating and reference sites is highly similar, indicating that rehabilitating activities after iron ore mining in the Urucum massif can rehabilitate soil microorganisms successfully.

## Usage Notes

Contigs and the taxonomic and functional classifications have been generated using an automated process without manual assessment, i.e., represent a draft assembly only. As such, all downstream research should independently assess the accuracy of reads, contigs, and taxonomic and functional assignments for organisms of interest. Nevertheless, this study presents a baseline for further studies of this kind.

The dataset contains a significant amount of taxa and functions previously identified, but a high portion of unclassified or incompletely classified CDS indicates the presence of a sizable portion of unseen biodiversity within soils along the sampled rehabilitation chronosequence. The identification of this unseen biodiversity may require additional alignments, eventually using different genome assemblers as well as combinations with further reference databases. Furthermore, there is a need for manual assessment of the quality of functional and taxonomic classification in some cases. This analysis of outstanding seen and unseen biodiversity within this dataset is expected to produce helpful insights to microbial community ecology along rehabilitation chronosequences after iron ore mining.

## Additional information

**How to cite this article**: Gastauer, M. *et al.* A metagenomic survey of soil microbial communities along a rehabilitation chronosequence after iron ore mining. *Sci. Data*. 6:190008 https://doi.org/10.1038/sdata.2019.8 (2019).

**Publisher’s note**: Springer Nature remains neutral with regard to jurisdictional claims in published maps and institutional affiliations.

## Supplementary Material



## Figures and Tables

**Table 1:** Site information for all 13 sampling locations utilized in this study.

Category	Study sites	Sample Alias	Latitude	Longitude	Age
Non-revegetated study sites	Rampa Nova	NR_RN_1 – NR_RN_3	−19.1950	−57.6030	0
	Mina 5	NR_M5_1 – NR_M5_3	−19.1848	−57.6111	0
	PRAD 45 C	NR_PR_1 - NR_PR_3	−19.2171	−57.5908	0
Sites in environmental rehabilitation	Piscinão	RH_PI_1 - RH_PI_3	−19.1918	−57.6024	6
	Mina Cateto	RH_MC_1 - RH_MC_3	−19.2168	−57.5817	3
	PRAD 45 A	RH_PA_1 - RH_PA_3	−19.1855	−57.6075	3
	Mina Escarpa	RH_ME_1 - RH_ME_3	−19.1927	−57.6032	3
	Secção 10I	RH_SC_1 - RH_SC_3	−19.1909	−57.6020	2
	Mina 5 N	RH_M5_1 - RH_M5_3	−19.2178	−57.5864	2
	PRAD 45B	RH_PB_1 - RH_PB_3	−19.1840	−57.6110	2
Reference sites, covered by natural *canga* vegetation	Reference A	REF_A_1 – REF_A1_3	−19.1921	−57.6016	—
	Reference B	REF_B_1 – REF_B_3	−19.1837	−57.6126	—
	Reference C	REF_C_1 – REF_C_3	−19.2095	−57.5935	—
Age is the time interval (in years) between the beginning of rehabilitation activities and sampling.					

**Table 2:** Sequencing and assembly data from metagenomic libraries of 34 soil samples from non-rehabilitated, rehabilitating and reference sites from two iron-ore mines, Corumbá, Mato Grosso do Sul, Brazil.

Sample Alias	Sample ID	Date	Latitude	Longitude	Category	Age (year)	Forward reads																	Reverse reads			Estimated coverage	Assembly		
							# bases	# reads	interval	# bases	# reads	interval	# contigs	Total length (Mbp)	N50															
NR_M5_1	MG171	10/6/2016	−19.1848	−57.6111	non-rehabilitated	0	2.852 + E09	3.780 + E07	43–76	2.851 + E09	3.780 + E07	63–76	33.12%	135,797	99.14	1387
NR_M5_2	MG172	10/6/2016	−19.1848	−57.6111	non-rehabilitated	0	2.573 + E09	3.417 + E07	35–76	2.572 + E09	3.417 + E07	62–76	22.02%	110,156	61.22	943
NR_M5_3	MG173	10/6/2016	−19.1848	−57.6111	non-rehabilitated	0	3.657 + E09	4.855 + E07	35–76	3.658 + E09	4.855 + E07	58–76	23.52%	127,830	63.76	841
NR_PR_1	MG152	10/7/2016	−19.2171	−57.5908	non-rehabilitated	0	2.383 + E09	3.168 + E07	35–76	2.383 + E09	3.168 + E07	61–76	19.76%	48,854	22.46	801
NR_PR_2	MG153	10/7/2016	−19.2171	−57.5908	non-rehabilitated	0	2.118 + E09	2.812 + E07	35–76	2.117 + E09	2.812 + E07	60–76	14.69%	71,968	36.74	837
NR_PR_3	MG147	10/7/2016	−19.2171	−57.5908	non-rehabilitated	0	1.076 + E09	1.428 + E07	38–76	1.076 + E09	1.428 + E07	60–76	15.68%	15,018	6.33	670
NR_RN_1	MG141	10/5/2016	−19.195	−57.603	non-rehabilitated	0	3.122 + E09	4.138 + E07	35–76	3.122 + E09	4.138 + E07	45–76	29.57%	192,916	134.66	1346
NR_RN_2	MG142	10/5/2016	−19.195	−57.603	non-rehabilitated	0	2.250 + E09	2.987 + E07	35–76	2.251 + E09	2.987 + E07	60–76	20.58%	70,101	36.29	826
NR_RN_3	MG143	10/5/2016	−19.195	−57.603	non-rehabilitated	0	3.429 + E09	4.546 + E07	35–76	3.429 + E09	4.546 + E07	58–76	35.23%	204,263	151.16	1485
REF_A_1	MG163	10/4/2016	−19.1921	−57.6016	Reference	—	3.706 + E09	4.911 + E07	35–76	3.706 + E09	4.911 + E07	62–76	27.03%	256,960	170.89	1180
REF_A_3	MG165	10/4/2016	−19.1921	−57.6016	Reference	—	3.097 + E09	4.106 + E07	35–76	3.097 + E09	4.106 + E07	45–76	24.83%	63,656	25.65	650
REF_B_1	MG156	10/6/2016	−19.1837	−57.6126	Reference	—	2.619 + E09	3.479 + E07	35–76	2.620 + E09	3.479 + E07	61–76	17.68%	48,507	21.04	740
REF_B_3	MG158	10/6/2016	−19.1837	−57.6126	Reference	—	3.747 + E09	4.970 + E07	35–76	3.747 + E09	4.970 + E07	62–76	19.39%	142,828	70.43	781
REF_C_1	MG174	10/7/2016	−19.2095	−57.5935	Reference	—	1.753 + E09	2.326 + E07	35–76	1.753 + E09	2.326 + E07	61–76	21.23%	65,769	28.36	674
REF_C_2	MG175	10/7/2016	−19.2095	−57.5935	Reference	—	2.100 + E09	2.783 + E07	43–76	2.100 + E09	2.783 + E07	60–76	13.99%	44,973	19.36	660
REF_C_3	MG166	10/7/2016	−19.2095	−57.5935	Reference	—	3.248 + E09	4.327 + E07	35–76	3.251 + E09	4.327 + E07	60–76	21.77%	87,360	36.41	686
RH_M5_1	MG170	10/6/2016	−19.2178	−57.5864	Rehabilitating	2	2.902 + E09	3.848 + E07	35–76	2.902 + E09	3.848 + E07	61–76	21.57%	60,777	27.05	688
RH_M5_2	MG161	10/6/2016	−19.2178	−57.5864	Rehabilitating	2	2.143 + E09	2.843 + E07	35–76	2.142 + E09	2.843 + E07	59–76	20.06%	43,609	21.8	825
RH_M5_3	MG162	10/6/2016	−19.2178	−57.5864	Rehabilitating	2	2.403 + E09	3.189 + E07	36–76	2.403 + E09	3.189 + E07	61–76	18.97%	50,393	24.65	883
RH_MC_1	MG167	10/6/2016	−19.2168	−57.5817	Rehabilitating	3	2.604 + E09	3.463 + E07	35–76	2.604 + E09	3.463 + E07	61–76	12.39%	15,205	5.61	612
RH_MC_3	MG169	10/6/2016	−19.2168	−57.5817	Rehabilitating	3	2.517 + E09	3.338 + E07	35–76	2.517 + E09	3.338 + E07	61–76	21.86%	38,569	19.38	1095
RH_ME_1	MG137	10/6/2016	−19.1927	−57.6032	Rehabilitating	3	2.853 + E09	3.794 + E07	35–76	2.854 + E09	3.794 + E07	61–76	16.45%	54,573	21.49	625
RH_ME_2	MG138	10/6/2016	−19.1927	−57.6032	Rehabilitating	3	1.763 + E09	2.339 + E07	35–76	1.763 + E09	2.339 + E07	61–76	16.54%	23,548	9.23	632
RH_ME_3	MG139	10/6/2016	−19.1927	−57.6032	Rehabilitating	3	3.668 + E09	4.874 + E07	35–76	3.669 + E09	4.874 + E07	60–76	19.06%	102,373	43.94	689
RH_PA_1	MG159	10/7/2016	−19.1855	−57.6075	Rehabilitating	3	2.916 + E09	3.881 + E07	35–76	2.914 + E09	3.881 + E07	61–76	25.52%	117,602	50.86	699
RH_PA_3	MG151	10/7/2016	−19.1855	−57.6075	Rehabilitating	3	2.505 + E09	3.322 + E07	36–76	2.504 + E09	3.322 + E07	61–76	18.39%	52,570	25.35	745
RH_PB_2	MG155	10/7/2016	−19.184	−57.611	Rehabilitating	2	1.748 + E09	2.321 + E07	35–76	1.748 + E09	2.321 + E07	60–76	20.37%	62,923	27.53	700
RH_PB_3	MG146	10/7/2016	−19.184	−57.611	Rehabilitating	2	2.336 + E09	3.101 + E07	35–76	2.335 + E09	3.101 + E07	62–76	16.42%	28,526	11.51	648
RH_PI_1	MG144	10/4/2016	−19.1918	−57.6024	Rehabilitating	6	2.609 + E09	3.461 + E07	35–76	2.608 + E09	3.461 + E07	60–76	13.79%	50,794	24.5	831
RH_PI_2	MG145	10/4/2016	−19.1918	−57.6024	Rehabilitating	6	2.450 + E09	3.251 + E07	35–76	2.451 + E09	3.251 + E07	61–76	17.47%	45,768	18.79	652
RH_PI_3	MG136	10/4/2016	−19.1918	−57.6024	Rehabilitating	6	2.045 + E09	2.728 + E07	35–76	2.049 + E09	2.728 + E07	60–76	11.01%	9,777	3.54	609
RH_SC_1	MG148	10/5/2016	−19.1909	−57.602	Rehabilitating	2	3.455 + E09	4.582 + E07	35–76	3.455 + E09	4.582 + E07	61–76	16.49%	49,559	24.64	778
RH_SC_2	MG149	10/5/2016	−19.1909	−57.602	Rehabilitating	2	1.606 + E09	2.129 + E07	35–76	1.606 + E09	2.129 + E07	62–76	16.57%	19,921	8.82	724
RH_SC_3	MG150	10/5/2016	−19.1909	−57.602	Rehabilitating	2	3.560 + E09	4.721 + E07	35–76	3.560 + E09	4.721 + E07	60–76	20.57%	95,547	48.65	761
N50 is an assembly statistics which indicates the length of the smallest contig in the smallest set of contigs whose total number of bases corresponds to at least 50% of the total length of the assembly^42^.																

**Table 3:** Taxonomic and functional classification of communities from metagenomic libraries of 34 soil samples from non-rehabilitated, rehabilitating and reference sites from two iron-ore mines, Corumbá, Mato Grosso do Sul, Brazil.

Sample Alias	Sample ID	Number of contigs	Number of CDS	Classified CDS	Unclassified CDS	Number of Genera	Number of different functions
NR_M5_1	MG171	135,797	123,230	113,675	9,555	1,664	6,769
NR_M5_2	MG172	110,156	88,340	78,069	10,271	1,652	6,057
NR_M5_3	MG173	127,830	84,564	76,370	8,194	1,583	6,027
NR_PR_1	MG152	48,854	34,574	28,374	6,200	1,467	4,498
NR_PR_2	MG153	71,968	53,781	44,922	8,859	1,586	51,59
NR_PR_3	MG147	15,018	10,461	9,283	1,178	853	2,741
NR_RN_1	MG141	192,916	163,614	145,797	17,817	1,808	7,540
NR_RN_2	MG142	70,101	54,039	48,130	5,909	1,544	5,262
NR_RN_3	MG143	204,263	175,879	157,329	18,550	1,824	7,324
REF_A_1	MG163	256,960	197,062	159,536	37,526	1,894	8,643
REF_A_3	MG165	63,656	39,039	30,236	8,803	1,428	5,195
REF_B_1	MG156	48,507	33,925	28,716	5,209	1,403	4,425
REF_B_3	MG158	142,828	191,768	84,886	16,882	1,737	5,794
REF_C_1	MG174	65,769	43,759	36,997	6,762	1,372	4,497
REF_C_2	MG175	44,973	31,717	27,736	3,981	1,261	4,149
REF_C_3	MG166	87,360	54,934	45,323	9,611	1,523	4,750
RH_M5_1	MG170	60,777	44,591	38,495	6,096	1,500	4,759
RH_M5_2	MG161	43,609	32,799	28,893	3,906	1,346	4,639
RH_M5_3	MG162	50,393	35,744	30,963	4,781	1,373	4,661
RH_MC_1	MG167	15,205	9,708	8,308	1,400	942	2,886
RH_MC_3	MG169	38,569	26,907	22,408	4,499	1,334	4,565
RH_ME_1	MG137	54,573	36,071	31,285	4,786	1,239	4,212
RH_ME_2	MG138	23,548	161,377	14,221	1,916	913	3,222
RH_ME_3	MG139	102,373	60,377	49,696	10,681	1,590	5,003
RH_PA_1	MG159	117,602	81,492	70,877	10,615	1,643	5,653
RH_PA_3	MG151	52,570	38,612	33,140	5,472	1,460	4,656
RH_PB_2	MG155	62,923	45,304	37,582	7,722	1,431	4,219
RH_PB_3	MG146	28,526	19,291	16,603	2,688	1,188	3,873
RH_PI_1	MG144	50,794	37,158	33,510	3,648	1,246	5,184
RH_PI_2	MG145	45,768	30,553	25,689	4,364	1,222	4,189
RH_PI_3	MG136	9,777	6,045	4,920	1,125	739	2,233
RH_SC_1	MG148	49,559	31,403	28,178	3,225	1,276	4,489
RH_SC_2	MG149	19,921	14,859	13,023	1,836	1,049	3,387
RH_SC_3	MG150	95,547	73,395	62,663	19,732	1,638	5,503
CDS are protein-coding sequences. The number of genera corresponds the number of distinct, fully identified genera of archaea, bacteria, and viruses.							

**Table 4:** Exclusive and core taxa for each sample cluster build with MGCOMP.

Cluster Id	Samples	Exclusive taxa	Core taxa
A	RH_ME_1	Subcluster 1: Frateuria, Leifsonia, Rhodanobacter, Dyella, RubrobacterSubcluster 2: Nonomuraea, Nocardiopsis, Microbispora, Thermomonospora, Actinopolymorpha	Anaeromyxobacter, Arthrobacter, Blastococcus, Chitinophaga, Flavihumibacter, Flavisolibacter, Frankia, Gemmatimonas, Gemmatirosa, Geodermatophilus, Janthinobacterium, Marmoricola, Massilia, Mucilaginibacter, Mycobacterium, Myxococcus, Niabella, Niastella, Nocardioides, Novosphingobium, Pedobacter, Phycicoccus, Ramlibacter, Segetibacter, Sinomonas, Sphingobium, Sphingomonas, Variovorax
	RH_ME_2		
	RH_PB_2		
	RH_PA_1		
	NR_M5_3		
	REF_C_1		
	REF_C_2		
B	RH_PI_3	Subcluster 1: Duganella, Lactococcus, Bryobacter, Chryseobacterium, Steroidobacter, Verrucomicrobium, Streptococcus, Lysobacter, Enterobacter, Geobacter, Belnapia, DechloromonasSubcluster 2: Microvirga, Pseudolabrys, Bosea, Rhodovulum	
	RH_ME_3		
	NR_RN_2		
	RH_PI_1		
	RH_PI_2		
	RH_PB_3		
	NR_PR_3		
	RH_SC_1		
	RH_SC_2		
	RH_SC_3		
	RH_PA_3		
	NR_PR_1		
	NR_PR_2		
	REF_B_1		
	REF_B_3		
	RH_M5_2		
	RH_M5_3		
	REF_A_3		
	REF_C_3		
	RH_MC_1		
	RH_MC_3		
	RH_M5_1		
C	NR_RN_1		
	NR_RN_3		
	NR_M5_1		
	NR_M5_2		
D	REF_A_1	Phenylobacterium, Caulobacter	
